# Production, purification, and characterization of cold-active lipase from the psychrotroph *Pseudomonas* sp. A6

**DOI:** 10.1007/s42770-023-01079-y

**Published:** 2023-08-02

**Authors:** Bahaa Abdella, Asmaa Mohamed Youssif, Soraya A. Sabry, Hanan A. Ghozlan

**Affiliations:** 1 Faculty of Aquatic and Fisheries Sciences, Kafrelsheikh University, Kafrelsheikh, 33516 Egypt; 2 Botany & Microbiology Department, Faculty of Science, Alexandria University, Alexandria, 21511 Egypt

**Keywords:** Lipase, Cold-adapted, Plackett-Burman, Optimization, Psychrotolerant

## Abstract

Cold-active lipases are presently employed extensively in the detergent, chemical intermediate, fine chemical, food, and pharmaceutical industries. Seven cold-adaptive bacteria were isolated from the Mediterranean Sea near Alexandria, Egypt, and tested for their ability to produce cold-active lipase, with the highest activity at 10 °C. The most potent isolate was *Pseudomonas* sp. A6. To determine the most important variables, the bacterium was exposed to a necessary medium component and environmental factor screening using a single factor-at-a-time approach, followed by a multifactorial Plackett-Burman design strategy. After purification and characterization, the optimal activity levels for the cold-active lipase were figured out. Inoculation of *Pseudomonas* A6 under near optimum conditions using medium consisting of (g/L) peptone 7.14; soybean oil 7.5% (v/v); K_2_HPO_4_, 0.4; MgSO_4_, 0.1; glucose 2; pH 8; and temperature 10 °C led to a maximum lipase activity anticipated to be 23.36 U/mL. Purified lipase showed the best activity and thermal stability at a pH of 8 and a temperature of 10 °C. The *Pseudomonas* A6 lipase tolerated the monovalent ions, while greater valence ions did not.

**Supplementary Information:**

The online version contains supplementary material available at 10.1007/s42770-023-01079-y.

## Introduction

The marine environment is rich in psychrotolerant bacteria. These bacteria have developed numerous adaptation mechanisms to help them withstand the severe impacts of such conditions [1, 2]. Lipases are hydrolytic enzymes that belong to the triacylglycerol hydrolases (EC: 3.1.1.3) family [3]. Even though lipases have been extensively investigated, cold-active lipases have not received much attention [4, 5]. Bacteria that can live at low temperatures generally have cold-active lipases [6]. Cold-adapted lipases are attractive biocatalysts in biotechnology because they are used as food additives or in laundry detergents to enable efficient washing at low temperatures [7]. They are also gaining popularity as a tool for producing extremely unstable compounds at low temperatures in the organic synthesis of chemical intermediates [6]. Moreover, in the manufacturing of fine chemicals, as well as in the food and pharmaceutical industries [8]. Cold-adapted was successful in catalyze the production butyl and oleic esters synthesis which has a bright future in biofuel and food industries [9, 10]. Microbial lipases had a $400 million market in 2017, and it is expected to increase to $590 million by 2023 [8]. Cold-active lipases are primarily produced extracellularly, making them suitable for fermentation and downstream purification processes. However, the physiology of the producing strain, environmental factors, and nutritional components such as carbon and nitrogen sources, inducer presence or absence, and so on are affecting the enzyme production [11, 12]. For instance, the type and concentration of carbon and nitrogen sources, as well as the aeration and pH value of the growth medium, are all factors that influence the lipase production [13]. The Plackett-Burman design was used before for screening of the most crucial factors in a biological process [14]. The isolation source is crucial to isolate psychrophilic microbes and is usually isolated from high altitude or from cold environments. However, the distribution of these microbes is not limited to such environments. Due to the change in the global weather, Alexandria city, Egypt, has experienced unusual cold weather in the winter during the past few years. This might have influenced the microbial structure in this geographical area. In this study, we successfully isolated a cold-adaptive *Pseudomonas* strain from the Mediterranean Sea. The growth requirements and production of cold-active lipase were examined using a multifactorial approach to figure out the major components that govern the lipase production. Furthermore, the cold-active lipase produced was purified and characterized for maximum effectiveness.

## Materials and methods

### Isolation of psychrotolerant bacteria and screening for cold-active lipase production

Seawater samples were collected following the method described by Arayes et al. [15] from different depths in clean sterile screw cap bottles. Bottles with a capacity of 250 mL were opened 15 cm and 1 m below the water’s surface in the Mediterranean Sea in Alexandria, Egypt, beside the National Institute of Oceanography and Fisheries (31°12′44.7″N 29°53′07.2″E). The water temperature was 22 °C. Within 4 h of the collection, samples were transported to the laboratory at 4 °C and processed. For isolation, seawater medium (SWM) was used, which included the following ingredients expressed in g/L: peptone 5; yeast extract 2.5; glucose 1; K_2_HPO_4_, 0.2; MgSO_4_, 0.05; and agar-agar 15. The components were dissolved in 75% of seawater, pH 7.2, and an antifungal agent, Mycostatin (1 mL/L), was added. SWM plates were surface inoculated with 1 ml of water samples and incubated at 10 °C for 3 to 7 days [16]. Colonies that grew on agar plates were picked up with a bacteriological needle and purified on the same medium using the traditional spatial streaking method. The pure bacterial isolates were then tested for their ability to grow in liquid SWM at pH 7 for 2 days while shaking at different temperatures (5 °C, 10 °C, 20 °C, and 30 °C). The isolates that showed high growth at 10 °C were selected for screening of lipase production. The screening was carried on SWM having 0.2% tributyrin, 1% Arabic gum, and 2% (w/v) agar [17]. After 7 days of incubation, a clear zone around the growth was considered a positive result.

### DNA extraction and molecular identification

The genomic DNA of the chosen isolate was extracted from a 2 mL of overnight bacterial culture using the technique outlined by Sambrook et al. [18]. The 16s rDNA was amplified using universal primers for 16s rRNA gene. The primers used: F27 (5′-AGAGTTTGATCMTGGCTCAG-3′) and 1492R (5′-TACGGYTACCTTGTTACGACTT-3′) [19]. In 50 μL PCR reaction buffer, 30 picomoles of each primer, 10 μL of chromosomal DNA, 200 mg dNTPs, and 2.5 units of Taq polymerase were mixed. The PCR was carried out for 30 cycles at 94 °C for 1 min, 55 °C for 1 min, and 72 °C for 2 min. The product was purified using a QIAquick (Qiagen) PCR purification kit following the manufacturer’s instructions, after the size of the PCR product was confirmed using 1% agarose gel electrophoresis. DNA sequences were obtained using an ABI PRISM 377 DNA Sequencer. Unipro UGENE integrated bioinformatics software was used to edit the obtained sequences [20]. The sequence was BLASTed against the NCBI non-redundant nucleotide database and the phylogenetic relationships were constructed using MEGA X [21].

### Culture conditions for lipase production

Seed culture (5 mL OD_600_=0.8–1.0) was inoculated into a 250-mL Erlenmeyer flask having 50 mL of SWM broth (pH 7) and incubated at 10 °C for 3 days under shaking conditions (160 rpm). For growth curve monitoring, samples were taken every 2 h to measure the optical density at 600 nm. After centrifugation at 10,000 g for 10 min, the cell-free culture supernatant was used to measure lipase activity.

### Lipase activity assay

The breakdown of *p*-nitrophenyl laurate and liberation of *p*-nitrophenol was measured spectrophotometrically at 420 nm [22]. Briefly, 0.1 mM phosphate buffer and *p*-nitrophenyl laurate were freshly prepared in ethanol. Then, 700 μL phosphate buffer and 100 μL of *p*-nitrophenyl laurate solution were added to 50 μL of the cell-free extract. After 30 min at 10 °C, 250 μL of Na_2_CO_3_ was added and centrifuged for 20 min at 13,000 rpm. The optical density of the resulting supernatant was measured at 420 nm against a blank. The blank was prepared using distilled water instead of cell-free supernatant. A standard curve was prepared using standard solutions based on the weight of pure *p*-nitrophenol ester. Under test conditions, one unit of lipase activity was defined as the quantity of enzyme that released one micromole of *p*-nitrophenol per min per milliliter.

### Factors affecting lipase production

The effects of several organic nitrogen sources (beef extract, yeast extract, malt extract, and peptone) on lipase synthesis were investigated either individually single-factor-at-a-time approach or in combination. The potential nitrogen source(s) were then tested for lipase production along with different oil types. The different oils used include coconut, soybean, sesame, castor, mustard, flaxseed, sunflower, olive, or deep-frying waste supplemented to the screening medium in 1.5% v/v. Over and above, glucose, fructose, and sucrose were studied as the only carbon source. In the optimization stage, the best carbon and nitrogen sources with the greatest lipase activity were used.

### Statistical experimental design

The Plackett-Burman experimental design of seven independent variables established the importance of medium components. The matrix in Table 1 shows that the seven independent factors resulted in 8 distinct combinations in 8 separate trials. Besides, the basal level at trial number 9 (Table 2). Matrix was created using the statistical software Statistica v6.0 (StatSoft Inc., 2001, USA). All trials were performed in triplicates, and the arithmetic mean of the triplicates was calculated as the response. The following equation was used to calculate the main effect of each variable:

$$\boldsymbol{Main}\ \boldsymbol{effect}=\frac{\sum R(H)-\sum R(L)}{N}$$

**Table 1:** Plackett-Burman design factors and their high and low values

Factors	Unit	Levels		
		Low (−)	Basal medium (0)	High (+)
Peptone	g/L	7.14	14.35	28.75
K_2_HPO_4_	g/L	0.1	0.2	0.4
MgSO_4_	g/L	0.025	0.05	0.1
Glucose	g/L	0.5	1	2
Soybean oil	mL/L	7.5	15	30
pH		5	7	9
Inoculum size	mL/flask	2.5	5	10

**Table 2:** The Plackett-Burman matrix with seven factors

Trial	Peptone	Soybean	K_2_HPO_4_	MgSO_4_	Glucose	pH	Inoculum size	Lipase activity (U/mL)
1	−1	−1	−1	1	1	1	−1	12.1
2	1	−1	−1	−1	−1	1	1	0.8
3	−1	1	−1	−1	1	−1	1	0.5
4	1	1	−1	1	−1	−1	−1	0.6
5	−1	−1	1	1	−1	−1	1	0.4
6	1	−1	1	−1	1	−1	−1	0.7
7	−1	1	1	−1	−1	1	−1	0.6
8	1	1	1	1	1	1	1	18
9	0	0	0	0	0	0	0	14.6

*R*(*H*) is the response parameter that holds a higher quantity of a given component. *R*(*L*) is the response parameter that holds a lower quantity of a given component. *N* is the number of combinations divided by 2. The *t*-test was used to calculate *t*-values, *p*-values, and confidence-level percentages for the experimental variables using Microsoft Excel.

### Purification of extracellular lipase

To purify lipase, various saturation levels of ammonium sulfate were added to the culture filtrate at 10 °C to precipitate the crude enzyme from cell-free supernatant. The added concentration was increased in ten percent increments from 20 to 100% saturation, then centrifuged for 15 min at 10,000 g at 10 °C. The precipitate was dialyzed against sterile distilled water after being dissolved in a 5 mM phosphate buffer at a pH of 7.0. The dialyzed protein was then purified using Sephadex G-100 column gel filtration chromatography. At a flow rate of 1 mL/min, after the column purification was run, the fractions were collected in a 3-mL quantity. The lipase activity was measured quantitatively in each fraction [23].

### Characterization of purified lipase

The pH of pure lipase was profiled at various pH values (from pH 5 to pH 10). The enzyme (50 μL) was added to a reaction mixture having *p*-nitrophenyl laurate as substrate and phosphate buffer (50 mM, 700 μL) at each pH level. The reaction mixture was incubated at 10 °C for 10 min before the lipase activity was estimated. The reaction mixture was incubated at various temperatures (10 to 50 °C for 10 min) to determine the optimal temperature for lipase activity. The thermostability of lipase was examined by pre-incubating the enzyme at different temperatures [24]. The effect of varying substrate concentrations on the enzyme activity was also investigated by increasing the concentration of the substrate in the reaction mixture from 0 to 2 mg/mL. The influence of several cations on enzyme activity (Fe^3+^, Cu^2+^, Na^+^, K^+^, Mn^2+^, and NH_4_^+^) was investigated. The cations were added to the reaction mixture individually at a concentration of 1% w/v [25]. The lipase’s molecular mass was figured out by mixing 10–20 μg of the pure enzyme with 2x SDS-loading buffer, denatured for 5 min at 95 °C, cooled on ice, and then loaded into a 12% SDS-PAGE with a protein ladder. The gel was run in glycine buffer for 10 min at 10 mV before being raised to 25 mV for 1 h. After staining, the gel was removed from the glass plates and de-staining was performed.

## Results

### Cold-active lipase production by psychrotolerant bacteria

Seven bacterial isolates capable of growing at 10 °C were isolated directly from seawater. Only a strain named A6 displayed the greatest growth (OD600 ~ 1.3) at 10 °C after all recovered isolates were examined for their best growing temperature. The ability of A6 to produce lipase enzyme was assessed qualitatively using the clear zone formation method (Supplementary Material Fig. S1). As a result, this isolate was chosen for future research. The best growth was seen at 10 °C and pH 7–8, with lesser growth in the alkaline range (pH 9 and 10). The bacteria grew well in various NaCl concentrations (0–30%), with 15% providing the best results.

### Molecular identification

**Fig. 1:**
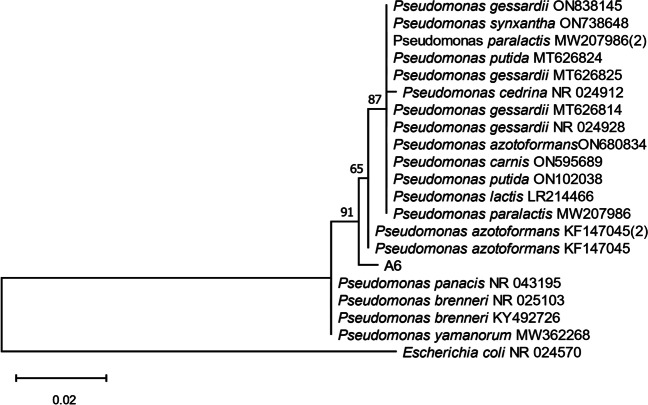
Phylogenetic relatedness of isolate A6. The tree was constructed using MEGA X with the closely related representatives of *Pseudomonas* species. *E. coli* is included as an outgroup. The tree was drawn to a scale showing the phylogenetic relationship among other *Pseudomonas* species

Based on phylogenetic relatedness analysis (Fig. 1), strain A6 was recognized as a novel member of the genus *Pseudomonas*. It is given the name *Pseudomonas* sp. A6. The nucleotide sequences were submitted to GenBank with the accession number KC417345. Blast revealed that it had a 100% similarity to the genus *Pseudomonas*.

### Factors affecting lipase production

**Fig. 2:**
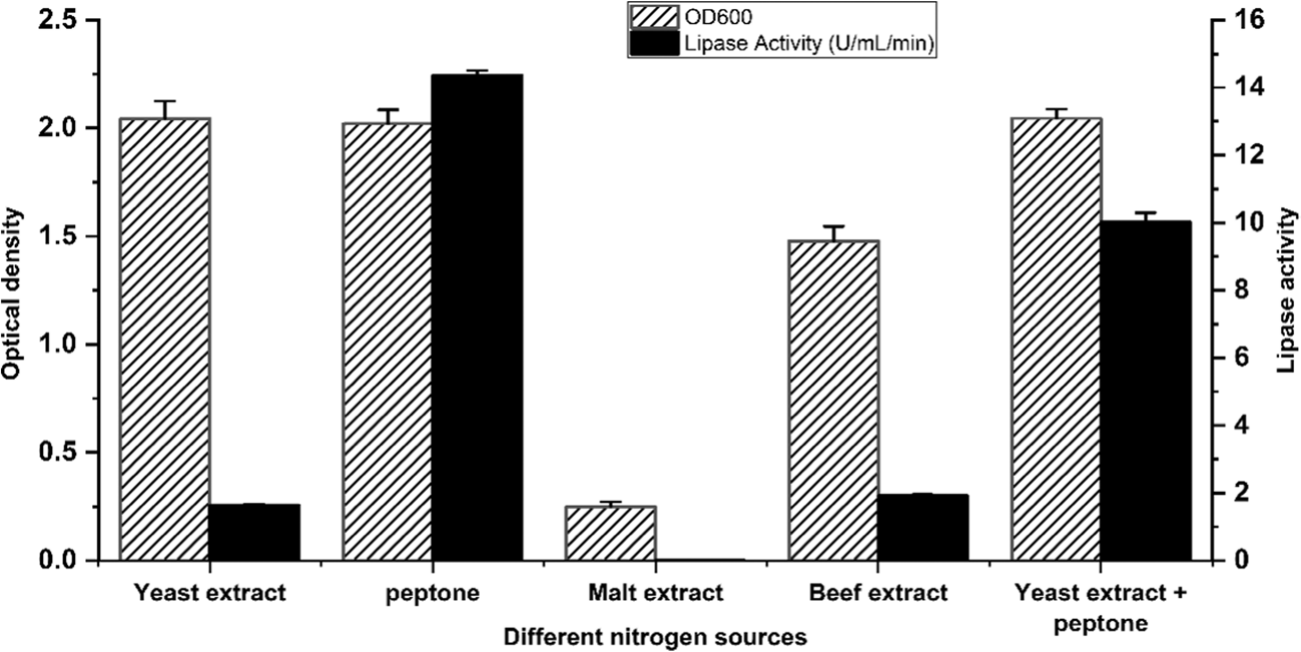
The effect of different nitrogen sources on the growth and lipase production. Error bars represent the standard error of mean (SEM) of the replica (*n*=3)

**Fig. 3:**
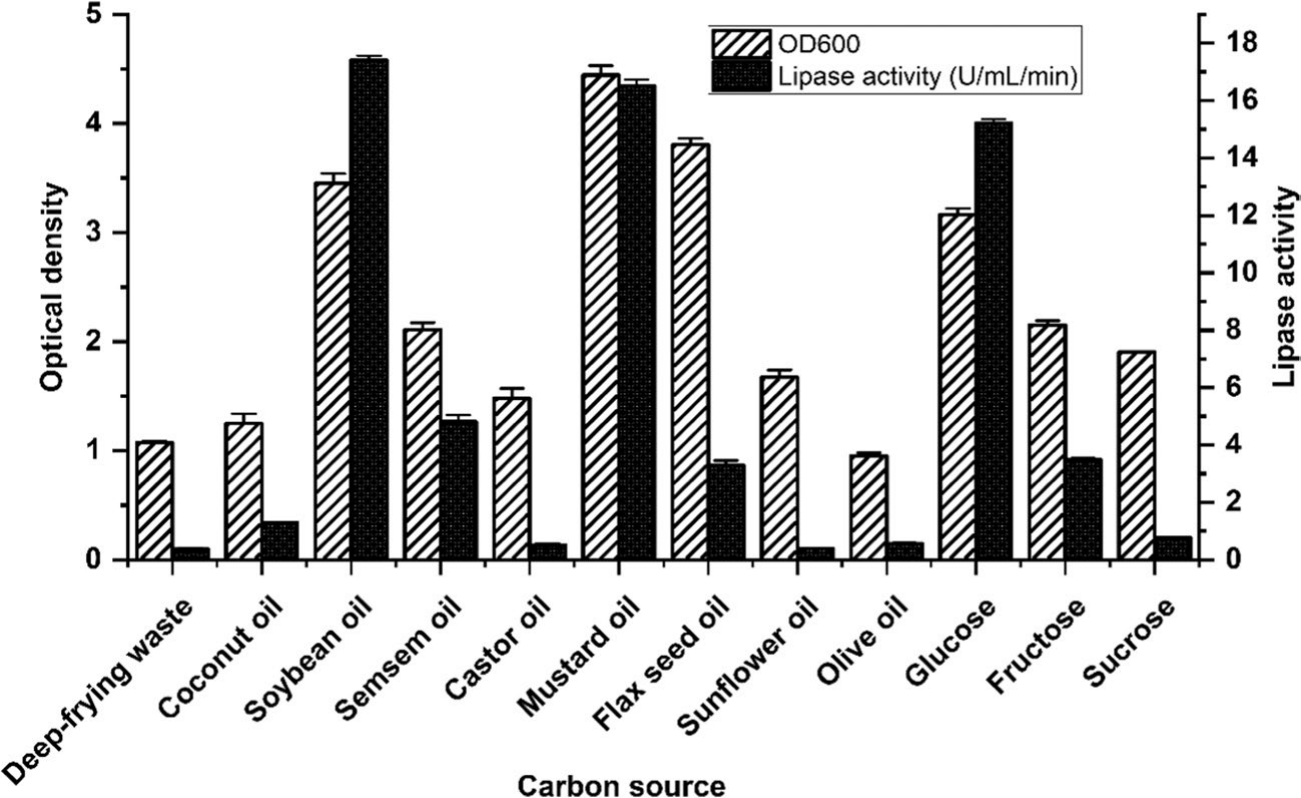
The effect of different oils and carbon sources on growth and lipase production. Error bars represent the standard error of mean (SEM) of the replica (*n*=3)

The greatest lipase activity (15 U/mL) was obtained when peptone was the only N source, whereas a combination of yeast extract and peptone yielded only 10U/mL (Fig. 2). The yeast extract, on the other hand, produced minimal activity (2 U/mL). In Fig. 3, glucose was the best carbon source among the simple sugars tested, with a lipase activity of 15 U/mL. Soybean oil, on the other hand, exhibited activity of 17 U/mL, which was greater than that of glucose among the oils examined.

### Plackett-Burman design for main effect determination

**Fig. 4:**
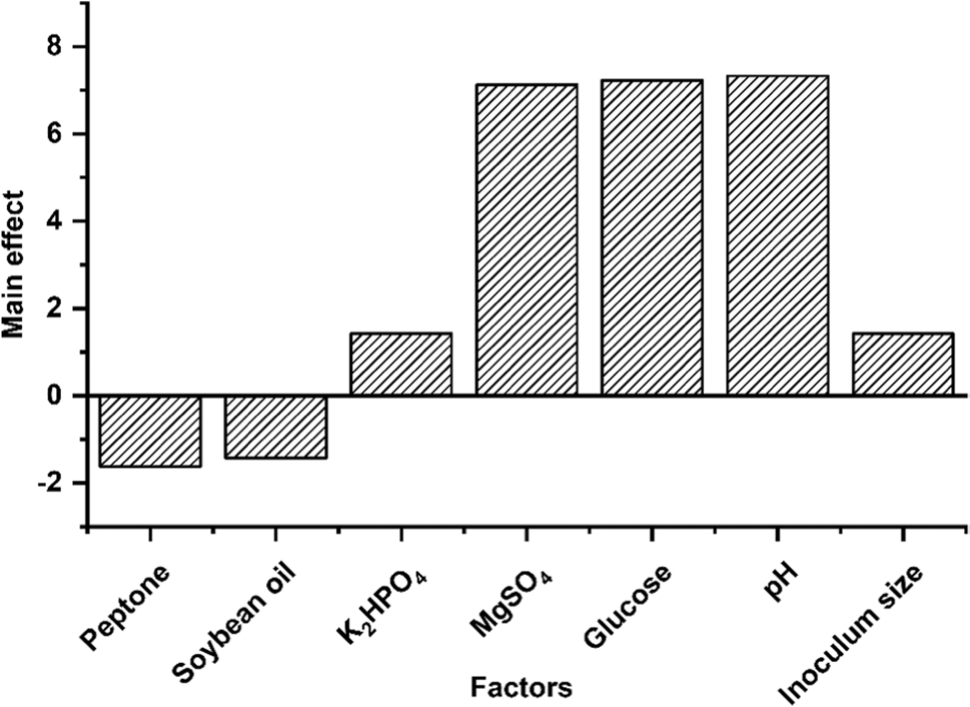
Elucidation of cultivation factors affecting *Pseudomonas* sp. A6 lipase production using Plackett-Burman experimental design

The main effect of the evaluated factors on lipase activity is estimated and visually shown in Fig. 4. The highly influential variables were MgSO_4_, glucose, and medium pH. The *t*-values, *p*-values, and confidence-level percentages for the experimental variables are shown in Table 3.

**Table 3:** Statistical analysis of the Plackett-Burman experimental results for *Pseudomonas* sp. A6

Factors	Lipase production			
	Main effect	*t*-value	*p*-value	Significance level (%)
Peptone	−1.625	0.258	0.45	54
Soybean oil	−1.425	0.212	0.5	50
K_2_HPO_4_	1.425	0.134	0.5	50
MgSO_4_	7.125	1.656	0.12	87
Glucose	7.225	1.686	0.12	87
pH	7.325	1.727	0.12	87
Inoculum size	1.425	0.207	0.47	52

**Fig. 5:**
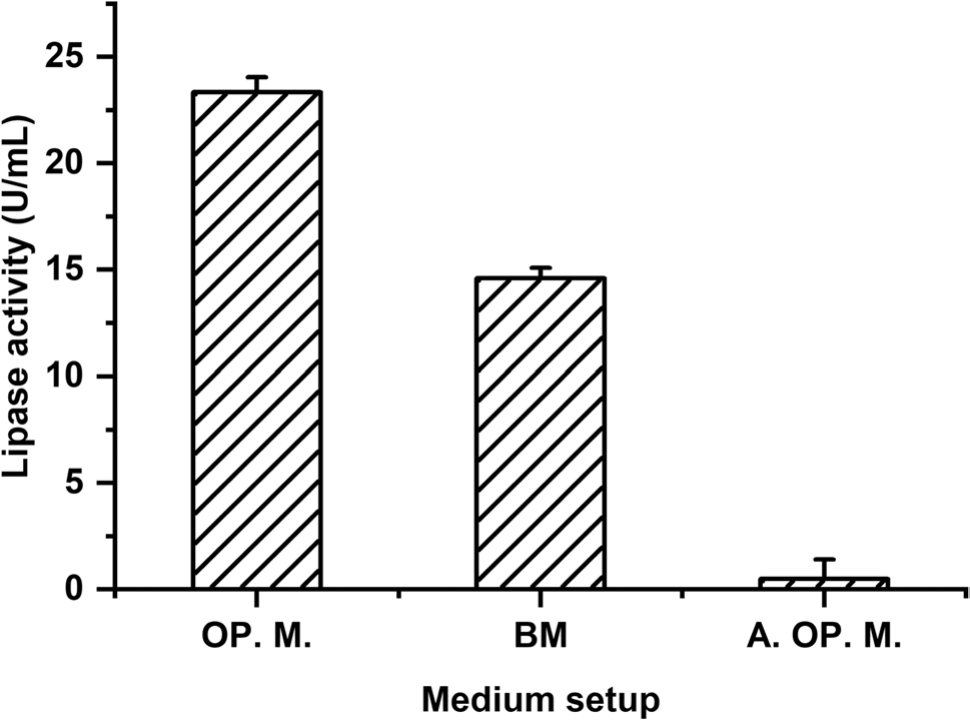
Verification experiments of the applied Plackett-Burman statistical design by comparing the lipase activity produced by *Pseudomonas* sp. A6 growing on the resulting optimized medium (OP.M), the basal medium (BM), and the anti-optimized medium (A.OP.M). Error bars represent the SEM of three replicas

The key factors boosting lipase synthesis, according to the main effect calculation for the factors under research and the confidence levels, were pH, glucose, and MgSO_4_. From here, a near optimum medium containing the following components (g/L): peptone 7.14; soybean oil 7.5% (v/v); K_2_HPO_4_, 0.4; MgSO_4_, 0.1; glucose 2; pH 8; temperature 10 °C; and incubation time 36 h should be about optimum. The basal, near optimal, and anti-optimum media were inoculated for verification, with the optimum medium showing a 1.5-fold increase in lipase activity to reach 23.36 U/mL. Lipase synthesis was completely suppressed in the anti-optimum experiments (Fig. 5).

### Cold-active lipase purification

**Fig. 6:**
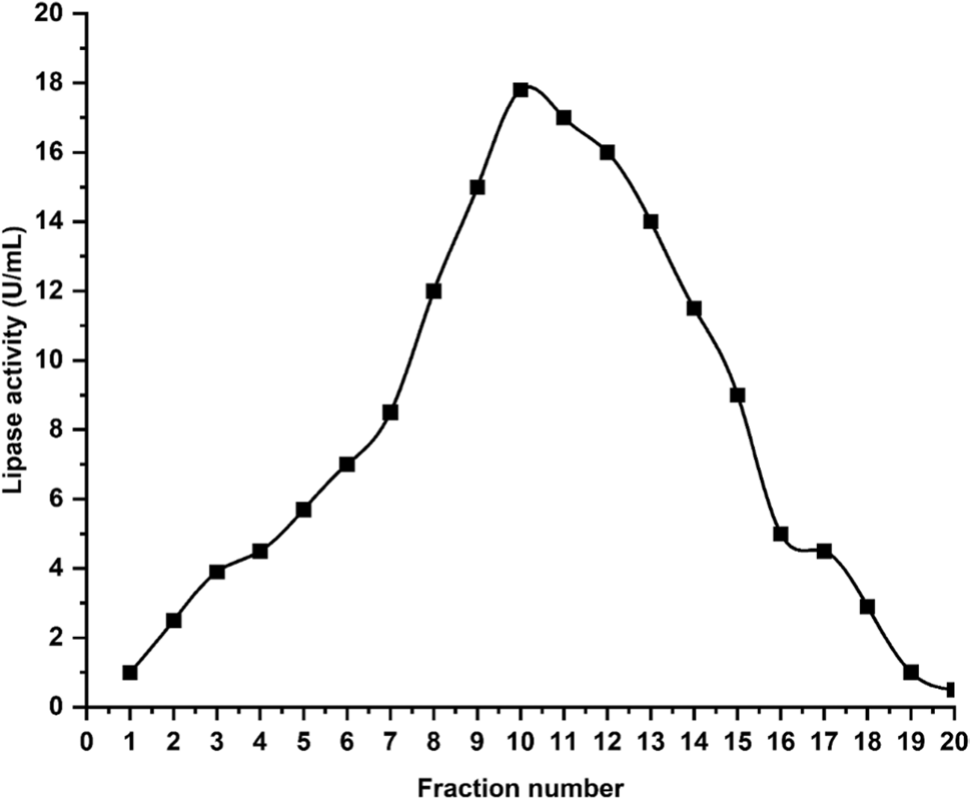
*Pseudomonas* sp. A6 lipase activity of different fractions obtained using Sephadex G-100

Purification of an enzyme is a key step in isolating the target protein and eliminating unwanted proteins. Extracellular lipase from *Pseudomonas* sp. A6 was isolated using a series of techniques. The purification profile of cold-active lipase is shown in Table 4. With a minor reduction of activity, the restored lipase had an activity of 17.8 U/mL. The recovered enzyme activity from each fraction is displayed in Fig. 6.

**Table 4:** Purification profile of cold-active lipase from *Pseudomonas* sp. A6

Purification step	Recovered volume (mL)	Total protein (mg)	Protein yield (%)	Activity (U/mL)	Total activity (U)	Specific activity (U/mg)	Purification fold
Speed centrifugation (crude enzyme)	800	40,000	100.00	23.36	18,688	0.467	1.0
Ammonium sulfate saturation 60%	60	1980	4.95	19.6	1,176	0.594	1.27
Gel filtration (Sephadex G-100)	10	200	0.5	17.8	178	0.89	1.91

### Cold-active lipase characterization

#### Effect of temperature and thermal stability

**Fig. 7:**
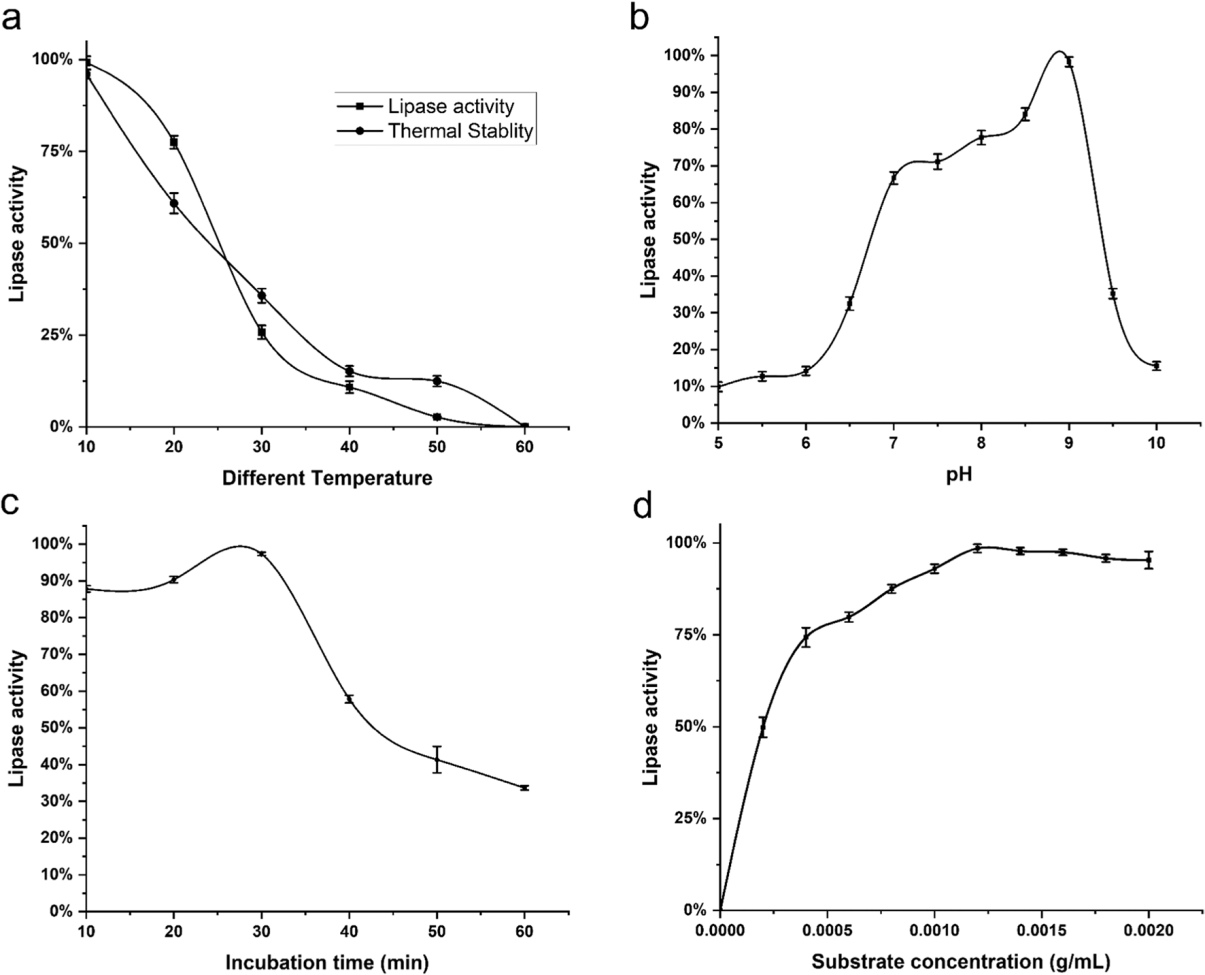
Effect of temperature and stability (**a**), different pHs (**b**), incubation time (**c**), and substrate concentration (**d**) on *Pseudomonas* sp. A6 purified cold-active lipase activity. Error bars represent the SEM of the replica (*n*=3)

Lipase activity of the *Pseudomonas* sp. A6 purified enzyme was figured out by incubating the reaction mixture (*p*-nitrophenyl laurate), purified lipase enzyme, and phosphate buffer, pH 7 at different temperatures (10 °C, 20 °C, 37 °C, and 50 °C). The maximum lipase activity was seen at 10 °C. Additionally, at 20°C, the cold-active lipase showed relatively high lipase activity (75% of its activity at 10 °C). It can also tolerate temperatures up to 30 °C with a considerable reduction in activity (Fig. 7a). The activity decreased abruptly with a further rise in temperature and was almost lost at 60 °C. The purified lipase was partially stable up to 50 °C but maximum stability was determined at 10 °C.

#### Effect of pH

At acidic pH (pH 6), the pure enzyme showed no activity. At pH 6.5, the activity was low, but it steadily rose with increasing pH, peaking at pH 9 at 10 °C. At pH 8.5–9, *Pseudomonas* sp. A6 lipase activity was at maximum (19.5 U/mL, 1 U/mg specific activity) (Fig. 7b).

#### Effect of incubation time

Pure lipase enzyme activity was measured by incubating the reaction mixture (*p*-nitrophenyl laurate, purified lipase enzyme, and phosphate buffer pH 9) at 10 °C for various durations of time (10 to 60 min). As shown in Fig. 7c, after 30 min of incubation, the maximum activity (19.7 U/mL and 1 U/mg) was detected. A longer incubation period, on the other hand, resulted in a considerable decrease in lipase activity.

#### Effect of substrate concentration

**Fig. 8:**
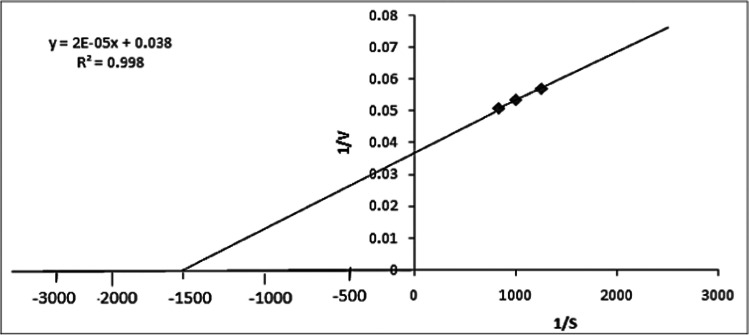
Determination of *K*_m_ expression of lipase activity

As showed in Fig. 7d, *Pseudomonas* sp. A6 lipase activity increased as substrate (*p*-nitrophenyl laurate) concentration increased up to a point of 0.0010 g/mL, after which the activity remained constant. The Michaelis constant (*K*_m_) was figured out from the Lineweaver and Burk plot (Fig. 8), by dividing the slope of the line by the intercept. *V*_max_ was figured out as the reciprocal of the intercept. The enzyme was found to have a *K*_m_ of 6.6 * 10^−4^ m mol L^−1^ and a *V*_max_ of 256.4 m mol L^−1^min^−1^.

#### Effect of enzyme concentration

The optimal enzyme concentration for maximal lipase activity (19.8 U/mL, 1.1 U/mg) by *Pseudomonas* sp. A6 was 1.4 mg/mL.

#### Effect of salts and metal ions

Metal ions can either promote or hinder the development of microbial enzymes. As shown in Table 5, Na+ ion was the most tolerable metal ion for lipase activity (19.5 U/mL, 1 U/mg), followed by K+ ion (16.6 U/mL, 0.85 U/mg), and NH4+ ion (15 U/mL). Alternatively, Cu^2+^ and Mn^2+^ inhibited the lipase activity.

**Table 5:** Effect of metal ions on *Pseudomonas* sp. A6 lipase activity

Metal ions and salt	Lipase activity (U/mL)
Control (no metal added)	19.7
Ferric ion	11.36
Copper ion	0.42
Ammonium ion	14.92
Manganese ion	0.38
Potassium ion	16.58
Sodium ion	19.5

#### Molecular weight determination of *Pseudomonas* sp. A6 cold lipase

**Fig. 9:**
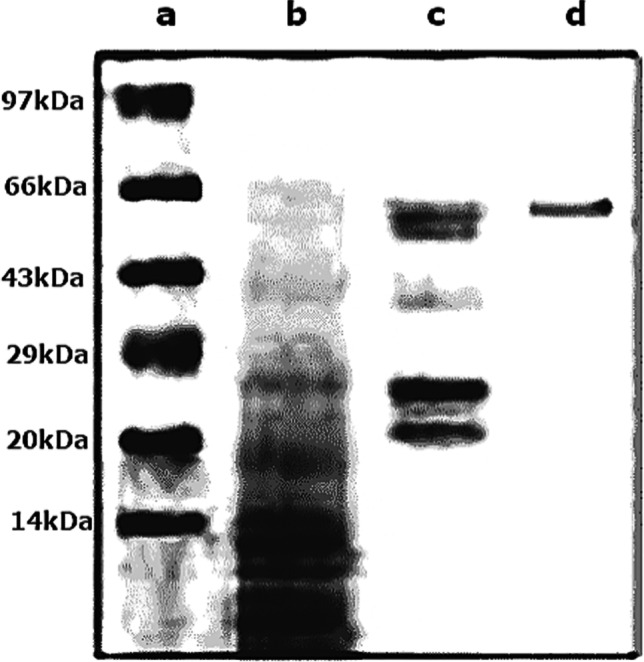
SDS-PAGE of purified lipase from *Pseudomonas* sp. A6: (**a**) protein marker, (**b**) crude enzyme, (**c**) partially purified enzyme by 60% ammonium sulfate, and (**d**) purified lipase

As shown in Fig. 9, the crude enzyme produced several bands, while the protein precipitated with 60% ammonium sulfate produced five bands. The molecular weight of lipase was 65 kDa in the purified fraction by column chromatography (Sephadex G-100).

## Discussion

Cold-adapted proteins and enzymes are found in psychrotolerant bacteria, which allow them to keep metabolic activity at low temperatures. Recently, many publications reported the production of cold-active lipase from *Pseudomonas* strains, for instance, *Pseudomonas fluorescence* KE38, *Pseudomonas* sp. LSK25, and *Pseudomonas* sp. CRBC14 [4, 26, 27]. An extracellular cold-adapted lipase enzyme from the marine psychrotolerant *Pseudomonas* sp. A6 that grew best at 10 °C was isolated and described in this work. *Pseudomonas* sp. A6 produced a novel cold-active lipase that was most active at 10 °C. A lipase with an optimal temperature and pH of 15 °C and 8, respectively, was isolated from the psychrotolerant *Pseudomonas* sp. AKM-L5 [28]. The lipase production in the current study showed maximum enzyme activity after 36 h of incubation during the stationary phase. Enzyme production is greatly dependent on the culture conditions like pH, temperature, and type of substrate. The highest growth for the organism was seen to be in the pH range of 7–8. Similar findings were seen for lipase production in neutral to alkaline media conditions by a psychrotrophic bacterium that was isolated from alpine regions [4, 29]. Moreover, lipases are produced by a variety of extremophiles, such as haloalkalitolerant [15]. A cold-active lipase was also discovered in another strain, *Pseudomonas* sp. AKM-L5 isolated from a soil sample in India. It has a molecular mass of 57 kDa as measured by SDS-PAGE, and a comparable optimal temperature of 10 °C to the lipase given, but pH 7 [29]. The optimum temperature and pH determined for lipase from *Pseudomonas* sp. KE38 were 25 °C and pH 8.5, respectively [27], which is considered high temperature and not a true cold-active lipase. On the other hand, and in accordance to our results, the lipase from *Pseudomonas* sp. LSK25 showed the exact optimum temperature of the reported lipase at 10 °C but lower pH value ranged from 7 to 7.5 [4].

Oil varieties used as lipase production inducers can affect enzyme production; soybean oil was produced the most, followed by mustard oil. Extracellular lipase synthesis is influenced by the kind of nitrogen supply as well as the carbon source. Peptone, an organic form of nitrogen, is extremely important since it supplies and serves as a precursor to the manufacture of vital amino acids, which are necessary for the formation of proteins, enzymes, and other cellular components [30]. The peptone and soybean were found to be the best nitrogen supply and oil substrate for generating cold-active lipase from *Pseudomonas* sp. A6. Their influence was not as strong or significant as the predicted main effect from the Plackett-Burman design. It is shown that MgSO_4_ and glucose concentration in the medium, in addition to pH value, was significant. The oil source is crucial as an inducer of lipase synthesis. In our study, soybean oil had the highest lipase activity compared to the other evaluated oil types.

In earlier research, olive oil was the best oil source for producing lipase from the fungus *Curvularia* sp. DHE 5 [31]. Using the Plackett-Burman optimization approach, MgSO_4_ was revealed to be essential to produce cold-active lipase from the *Bacillus cereus* HSS strain [12], which is consistent with our findings. In rodents and in vitro investigations, magnesium is important for lipase activity [32, 33]. Furthermore, pH 8 was identified as being truly relevant for lipase synthesis, which might be linked to the natural pH of seawater (pH 8.1) [34], from which the isolated strain was recovered and adapted to live in the marine environment. The best pH for cold-active lipase was in the alkaline range between pH 8 and 9.

Finally, the addition of glucose not only increased lipase synthesis in the presence of an oil substrate but also played a substantial role in the optimization experiment. It was reported that in the optimization of cold-active lipase from *B. cereus* HSS, the facile absorption of glucose to form the precursor necessary for microbial lipase may account for the favorable influence on lipase synthesis [12]; nevertheless, in a recent investigation, the presence of 20 mM glucose hindered lipase activity, which contradicts our findings [35].

Ammonium sulfate precipitation and gel filtering were used to efficiently purify *Pseudomonas* sp. A6 crude enzyme to homogeneity. Numerous investigations on the multi-step purification of cold-active lipase from various psychrophilic and psychrotrophic bacteria have been done. Ammonium salt precipitation is initially performed, followed by a succession of chromatographic phases [5, 11, 36].

SDS-PAGE revealed that the isolated lipase had a molecular weight of 65 kDa. The purified lipase’s biochemical evaluation revealed that it was active in the pH range of 7 to 9, with a best activity at pH 9. This shows that the purified lipase is alkaline. This form of alkaline active lipase has been shown in various microorganisms. Another alkaline lipase with activity in a pH range of 8.0–10.5 with maximum activity at pH 8.5 [37]. Such enzymes benefit from alkali tolerance since they are cold-active and actively stable in alkaline environments.

## Conclusion

The current study looked for cold-active lipase-producing bacteria from saltwater in the Alexandrian Mediterranean Sea. *Pseudomonas* sp. A6 is found to produce cold-active lipase. The Plackett-Burman statistical experimental approach was used to study the primary determinants regulating cold-active lipase. The optimum medium component found to be peptone 7.14 g/L; soybean oil 7.5% (v/v); K_2_HPO_4_, 0.4 g/L; MgSO_4_, 0.1 g/L; glucose 2 g/L; pH 8; and temperature 10 °C. The lipase produced under optimal culture conditions increased activity 1.5-fold when compared to the un-optimized medium. The features of the pure lipase enzyme generated have been carefully investigated, making it a practical option for various applications that need lipase activity at low temperatures.

## Supplementary information

ESM 1 (DOCX 469 kb)
