# Supplementary material for: Production, purification, and characterization of cold-active lipase from the psychrotroph Pseudomonas sp. A6
Source: Braz J Microbiol. 2023 Aug 2;54(3):1623–33. doi: 10.1007/s42770-023-01079-y (PMC10484855; doi:10.1007/s42770-023-01079-y)
Supplement: Supplementary file 1 — (DOCX 469 kb) [file 42770_2023_1079_MOESM1_ESM.docx]

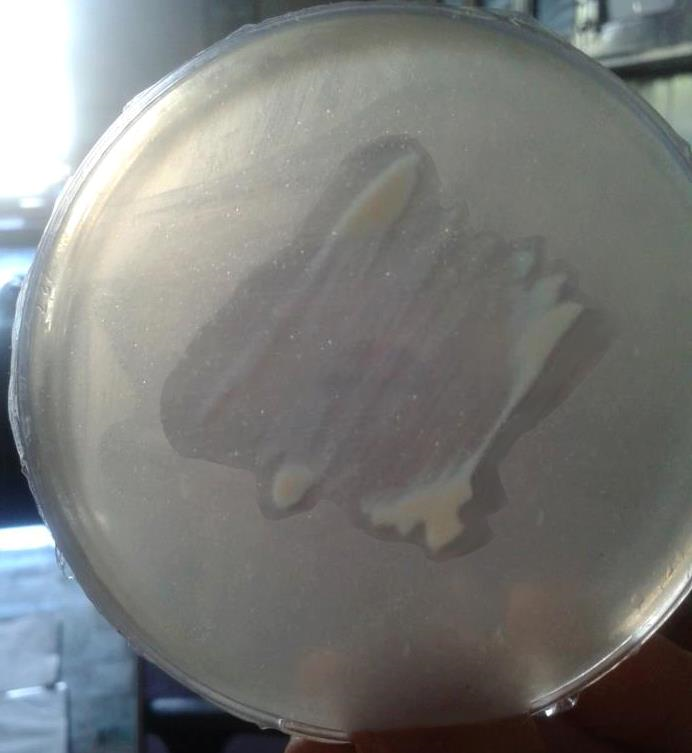


Fig 1S: Lipolysis of tributyrin by the psychrotroph *Pseudomonas* sp.A6 as detected by the formation of a clear zone around bacterial growth


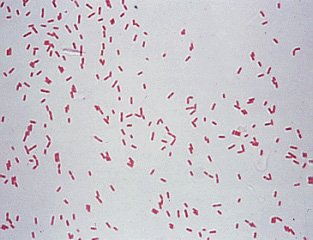


Fig 2S: Gram stain of *Pseudomonas* sp. A6 cells.


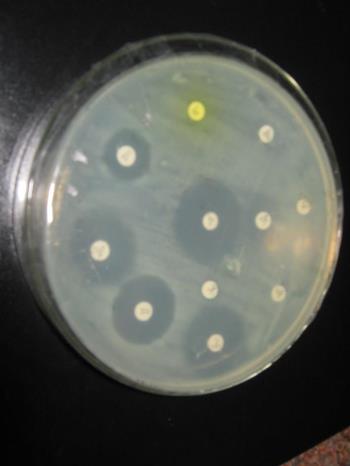


Fig 3S: Antibiotic sensitivity for *Pseudomonas* sp. A6 (SXT20): Sulfamethoxazole, (NOR10): Norfloxacin, (TOB15): Tobramycin, (RA10): Rifampin, (AMC10): Amoxicillin, (CEP5): Ceftazidime.

Fig 4S: Growth curve of *Pseudomonas* sp. A6 was grown in SWM and incubated at different temperatures under shaken conditions for 48 h.

Fig 5S: Effect of pH on growth of *Pseudomonas* sp. A6 was cultured in SWM and incubated at 10°C for 15 h under shaken conditions.

Fig 6S: Effect of NaCl concentration on the growth of *Pseudomonas* sp. A6 cultured in SWM (components dissolved in dist. water), pH 7, and incubated at 10°C for 36 h under the shaked condition
